# Prevalence and factors associated with renal dysfunction among children with sickle cell disease attending the sickle cell disease clinic at a tertiary hospital in Northwestern Tanzania

**DOI:** 10.1371/journal.pone.0218024

**Published:** 2019-06-18

**Authors:** Fransisca D. Kimaro, Shakilu Jumanne, Emmanuel M. Sindato, Neema Kayange, Neema Chami

**Affiliations:** 1 Department of Paediatrics and Child Health, College of Health Sciences - University of Dodoma, Dodoma, Tanzania; 2 Department of Internal Medicine, College of Health Sciences - University of Dodoma, Dodoma, Tanzania; 3 Department of Paediatrics, Catholic University of Health and Allied Sciences, Bugando Medical Center, Mwanza, Tanzania; University of Alabama at Birmingham, UNITED STATES

## Abstract

**Background:**

Little is known on how the interaction between Sickle Cell Disease (SCD) and renal insults caused by other coexisting conditions in Sub Saharan Africa such as urinary schistosomiasis, malnutrition and HIV affect the prevalence of renal dysfunction in children with SCD.

**Objectives:**

To determine the prevalence and factors associated with renal dysfunction among children with SCD aged 6 months to 12 years attended at a tertiary hospital in Northwestern Tanzania.

**Methods:**

A cross sectional hospital-based study with a short follow up component of 3 months for 153 children with SCD was done to document demographics, clinical characteristics and features of renal dysfunction including urine dipstick albuminuria (>20mg/l) and eGFR (<60ml/ml/min/1.73m^2^). Other potential renal insults such as HIV infection and Schistosomiasis were also evaluated.

**Results:**

At enrollment, 48/153(31.37%) children had renal dysfunction declining to 31(20.3%) at 3 months follow up. Acute chest syndrome (OR 3.04, 95% CI [1.08–8.96], p = 0.044), severe anemia (OR 0.44, 95% CI [0.26–0.76],p = 0.003), urinary schistosomiasis (OR 7.43, 95% CI [2.10–26.32] p<0.002) and acute malnutrition (OR 4.92, 95% CI [1.29–18.84], p = 0.020). were associated with renal dysfunction.

**Conclusion:**

Where prevalent, urinary schistosomiasis and acute malnutrition increase the risk for renal dysfunction in children with SCD. We recommend albuminuria routine screening in children with SCD especially those presenting with acute chest syndrome, severe anemia and features of acute malnutrition for early detection of renal dysfunction among children with SCD.

## Introduction

Progressive decline in renal function is a common occurrence among children with Sickle Cell Disease (SCD). This starts during infancy marked by reduced urine concentrating ability, glomerular hyperfiltration and moderately increased albuminuria culminating to severely increased albuminuria, Chronic Kidney Disease (CKD) and End Stage Renal Disease (ESRD) among adolescents and young adults[1]. Progressive renal dysfunction is an important attribute to the reduced life expectancy in SCD patients and is reported to contribute 16–18% of the overall mortality in this population[2]. Homozygous state (HbSS) and HbSβ^0^ thalasemia tends to have more severe renal involvement than other subtypes of sickle cell anemia such as HbSC and HbSβ^+^thalasemia[3].

The actual mechanisms leading to sickle cell related renal complications remain unclear but the hypoxic, hypertonic, and acidic environment in the renal medulla is postulated to induce sickling of red blood cells in the vasa recta leading to vaso occlusion, infarction and ischemia[4]. The resultant ischemic changes trigger the release of vasodilatory agents such as prostaglandins and nitric oxide causing increased renal blood flow and hyperfiltration[5]. Hyperfiltration is characterized by progressive elevation of GFR until adolescence when the GFR start to decline[6]. The defective urinary concentrating ability(hyposthenuria), hyperfiltration, albuminuria/proteinuria and hematuria eventually progress to reduced Glomerular Filtration Rate (GFR) and ESRD[7,8]. In addition, the chronic hemolysis in SCD produces free hemoglobin which increases the scavenging of nitric oxide through direct reaction and competitive reduction of arginine by arginase released by hemolyzed RBCs[9]. These complex interactions have been linked with the hemolysis induced endothelial injury leading to the vasculopathy seen in SCD acute and chronic complications such as nephropathy and pulmonary hypertension[10,11].

Little is known about the spectrum of renal dysfunction among SCD patients in Sub Saharan Africa where other potential nephrotoxic conditions such as malaria, schistosomiasis and HIV infections co-exist at high prevalence [12–14]. The prevalence of SCD in northwestern Tanzania (along Lake Victoria) is estimated to be around 1.4%. Previous studies have reported a high prevalence of renal dysfunction among children in this region and suggested significant association with SCD, underscoring the need for specific studies focusing on children with SCD to understand the magnitude of the problem and the contribution of other coexistent conditions on renal dysfunction in this population[15]. We conducted this study to determine the prevalence of renal dysfunction among children with SCD and the association of other endemic conditions to the occurrence of renal dysfunction in this population of patients.

## Material and methods

This was a cross sectional descriptive study with a short follow up component of 3 months post enrolment, conducted at Bugando Medical Center (BMC); a 900-beds tertiary referral hospital for the northwestern zone of Tanzania located in the city of Mwanza.The study was conducted from October 2015 to April 2016 enrolling children with SCD aged 6 months to 12 years who were attending the outpatient clinic for routine SCD care. Ethical Clearance was granted by the CUHAS-Bugando Ethical and Research review Committee and parents/guardians signed an informed consent form before enrollment of their children in our study.

Children were consecutively enrolled to the required sample size (80% power to determine statistical significance) if they had an electrophoresis confirmed HbSS disease and their caretakers/parents gave consent for enrollment. Children with acute Sickle cell crisis, fever (temperature > 38°C), urinary tract infection (on urine dipstick), malaria, and those who required inpatient care at enrolment were excluded from the study.

### Study procedures

Children attending the pediatric sickle cell clinic at BMC during the study period were screened for eligibility before enrollment. Written informed consents were obtained from parents or caretakers, and assent from children older than 7 years.

Those enrolled had their medical records reviewed by the study investigators for confirmed HbSS status and blood samples for hemoglobin electrophoresis were collected for those with unknown disease phenotype, and they were included in the study if they were HbSS phenotype. Questionnaires were used for collection of patient information including demographics, clinical details, medication use, frequency and causes of previous admissions. Physical examination was conducted to look for jaundice, edema, liver and spleen size, weight, height/length and WHO Z-score charts were used to assess nutritional status. Other features including vital signs (i.e. pulse rate, respiratory rate and blood pressure) were also taken. Body Mass Index (BMI) = weight (kg)/ [height (m)]^2^ was calculated for those above 5 years and interpreted according to WHO growth charts. For children below 5 years of age, WHO weight-for-height charts were used to determine the nutrition status and were classified as moderate or severe malnutrition if weight for height WHO Z-score were between -2 Standard Deviation (SD) and -3SD and less than-3SD respectively. Blood pressure measurements were taken three times by manual pressure cuffs matched for child’s forearm size and then averaged to calculate a final blood pressure.

### Urine sample collection and measurement for albuminuria

A spot clean catch or next day early morning urine (if unable to provide a spot sample) was collected in a sterile bottle by the caretakers, older children were instructed on urine collection techniques. At least 10mls of urine was collected in bottles labeled with participant’s code numbers and analyzed on the same day. The urine samples were first tested using the normal Uro-dip-10e strips (ERBAR Diagnostics, Mannheim, German) to detect features suggestive of urinary tract infections such as leukocyte esterase and nitrite to rule out false positive proteinuria. Samples were then analyzed by Micral-test strip (Roche Diagnostics, Basel Switzerland), test strips that detect albuminuria in spot urine sample with reported sensitivity and specificity of 100% and 86.6%, respectively[16]. Albuminuria was defined by varying shades of pink in the test strip that correspond to a range of 20–100 mg/L of urinary albumin. Schistosomiasis cathodic antigen (CCA) test (Rapid Medical Diagnostics; Pretoria, South Africa) was performed for all urine samples and those who tested positive were treated with oral Praziquantel.

### Blood measurements and eGFR estimation

Venous blood was taken using standard aseptic techniques. For serum creatinine, 2mls of blood was obtained and the required sample assay was conducted using a COBAS INTEGRA 400 machine (Roche Diagnostics, Basel Switzerland), employing the buffered kinetic Jaffe’ reaction. The serum creatinine results were used to calculate the eGFR using a modified pediatric Schwartz equation as follows: GFR = (k * height)/ Serum Creatinine where k = 0.45 for infants and 0.55 for those aged 1–12 years[17,18].

Additional 2mls of blood were obtained in EDTA containers for full blood count and hemoglobin electrophoresis for patients not previously diagnosed by hemoglobin electrophoresis. Hemoglobin level results were categorized using the WHO anemia severity classification as mild (Hb 10–10.9g/dl), moderate (Hb 7–9.9g/dl) and severe anemia (Hb <7g/dl).

HIV testing was performed in all study participants after counseling and random blood glucose test was performed to all children at enrollment. Those with a positive albuminuria and or eGFR<60mls/min/1.73m^2^ were followed-up for 3 months with a repeat urine sample for protein and serum creatinine tests for eGFR estimation to confirm persistent renal dysfunction.

Data were analyzed using Stata version 13 (Stata Corp, Texas, USA). Results were summarized using proportions (%) for categorical data and continuous variables were summarized as median & inter-quartile ranges.

The prevalence of renal dysfunction was determined by taking the number of children with renal dysfunction over the enrolment number. The predictors of renal dysfunction were determined by univariate, then multivariate logistic regression, odds ratio and 95% confidence interval, p-value of <0.05 was considered significant.

## Results

### Study enrolment

**Fig 1:**
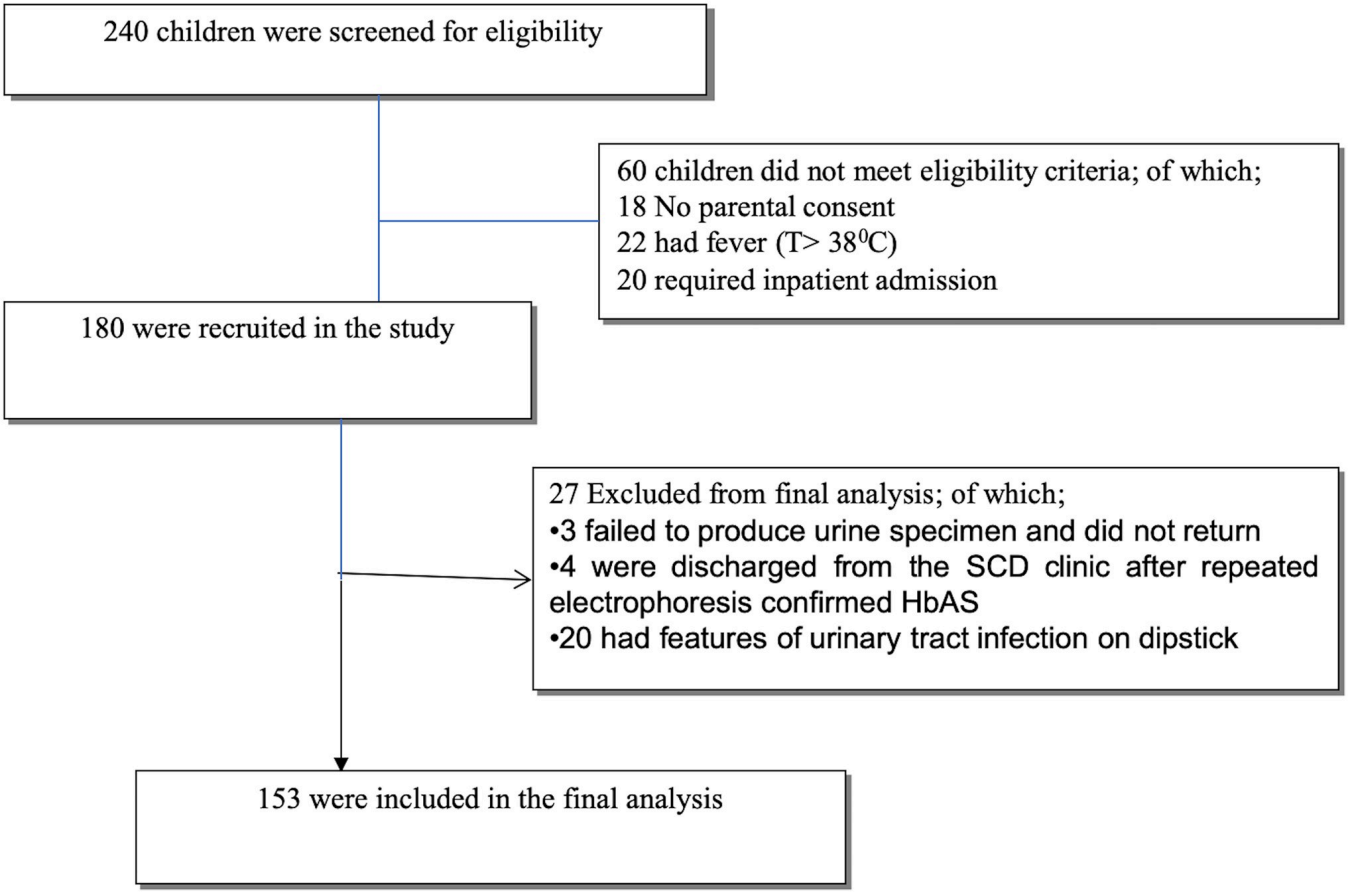
A consort diagram of the study enrollment break down.

During the study period; 240 children attended the SCD clinic at BMC and were screened for eligibility to be enrolled. One hundred and fifty-three (153) children were finally eligible for enrollment, 87children were not included in our current analysis due to various reasons as depicted in “Fig 1”.

### Patient characteristics

One hundred and fifty-one [151 (98.7%)] children were of African ethnicity and only 2 (1.3%) were of Asian descent with 88 (57.5%) being males. The median age of study participants was 72months with median age at SCD diagnosis of 30(12–60) months. Only 20(13.1%) children had their blood pressure taken at least once in the past one year prior to our study and at enrollment three (1.95%) children had history of elevated blood pressure but normalized in two weeks and none of them had renal dysfunction. Three children (1.9%) had history of reduced urine output, while 12 children (7.8%) were reported to have history of blood in urine, 6 children (3.9%) were on hydroxyurea therapy and none of them had renal dysfunction. Sustained NSAID use (2 weeks or more) was reported in 11 (7.2%) children, herbal medications use and traditional healer visits for SCD care was reported among 37(24.2%) and 80 (52.3%) children respectively. Schistomiasis test (using cathodic antigen) was positive in 21(13.7%) of children. All the study children were negative for HIV. Other baseline and clinical characteristics are presented in “Tables 1 and 2”.

**Table 1:** Baseline demographic characteristics of 153 children with SCD enrolled in the study.

Characteristic	Overall cohort(N = 153) Number (Percent)	Renal dysfunction(N = 31) Number (Percent)	No renal dysfunction(N = 122) Number (Percent)
***Gender***			
Male	88(57.5)	17(54.8)	71(58.2)
Female	65(42.5)	14(45.2)	51(41.8)
***Age categories***			
< 3 years	40(26.1)	3(9.7)	37(30.3)
3–6 years	34(22.2)	7(22.5)	27(22.1)
6.1–9 years	34(22.3)	10(32.3)	24(19.7)
9.1–12 years	45(29.4)	11(35.5)	34(27.9)
***Patient ethnicity***			
African	151(98.7)	31(100.0)	120(98.4)
Asian	2(1.3)	0(0.0)	2(1.6)
***Water source***			
Tap water	128(83.6)	26(83.9)	102(83.6)
Lake/pond water	25(16.4)	5(16.1)	20(16.1)
***Toilet***			
Flush toilet	119(77.8)	22(71.0)	97(79.5)
Pit latrine	34(22.2)	9(29.0)	25(20.5)
Average monthly income (TZS)*			
**<**500,000	106(69.2)	18(58.1)	88(72.1)
500,000 to 1,000,000	42(27.5)	13(41.9)	29(23.8)
>1,000,000	5(3.3)	0(0.0)	5(4.1)

*Tanzanian Shillings (Exchange rate at submission 1USD = 2246TZ

**Table 2:** Baseline clinical characteristics of 153 enrolled children with SCD.

Characteristic	Overall cohort(N = 153) Number/Median(%/IQR)	Renal dysfunction(N = 31) Number/Median(%/IQR)	No Renal dysfunction(N = 122) Number/Median(%/IQR)	p-value
***SCD related hospital admissions***	139(90.8)	28(90.3)	111(91.0)	0.909
***History of acute chest syndrome***	30(19.6)	10(32.3)	20(16.4)	0.047
***History of neurologic deficit***	5(3.3)	5(16.1)	0(0.0)	<0.001
***SCD related traditional healer visit***	62(40.5)	14(45.2)	48(39.3)	0.556
***Current use of herbal medicines***	32(20.9)	9(29.0)	23(18.9)	0.213
***History of blood in urine***	11(7.2)	7(22.6)	4(3.3)	<0.001
***Family history of kidney disease***	2(1.3)	2(6.5)	0(0.0)	0.005
***NSAID’s use for> 2 weeks in row***	11(7.2)	2(6.5)	9(7.4)	0.859
***Prior throat or skin infections***	23(15.0)	7(22.6)	16(13.1)	0.188
***Nutritional status***				0.004
Well nourished	87(56.8)	14 (45.2)	73 (59.8)	
Mild malnutrition	48(31.4)	8 (25.8)	40 (32.8)	
Moderate malnutrition	14(9.2)	8(25.8)	6(4.9)	
Severe malnutrition	4(2.6)	1(3.2)	3(2.5)	
***Positive Urine CCA test***	21(13.7)	11(35.5)	10(8.2)	<0.001
***eGFR (mL/min/1*.*73m***^***2***^***)***	142(107–221)	140(108–294)	143(103–220)	0.132
***Categories of hemoglobin level***				0.284
Mild anemia (Hb 10–10.9g/dl)	3(2.0)	1(3.2)	2(1.6)	
Moderate anemia (Hb 7.0–9.9g/dl)	111(72.5)	19(61.3)	92(75.4)	
Severe anemia (Hb <7gldl)	39(25.5)	11(35.5)	28(23.0)	

### Prevalence of renal dysfunction among children with SCD enrolled

Forty-eight (31.4%) of the study children met our definition for renal dysfunction (eGFR < 60 ml/min/1.73m^2^ and/or albuminuria of 20mg/l or more). At enrolment, more than a quarter of the children 44 (28.8%) had albuminuria and 4 (2.6%) had eGFR < 60ml/ *ml/min/1*.*73m*^*2*^. At three months follow up 45 children were available for re-evaluation and 31/45 had renal dysfunction making the prevalence of renal dysfunction among enrolled children to be 20.3% (31/153) and transient renal dysfunction was 9.2% (14/153). More details on the prevalence of Renal dysfunction among study children were as shown in “Table 3”.

**Table 3:** Prevalence of renal dysfunction among (153) children with HbSS enrolled in the study.

Variable	Number (n)/Median	Percent (%)/IQR
*eGFR (mL/min/1*.*73m*^*2*^*)*		
***eGFR categories***		
90+	127	83.0
60–89	22	14.4
30–59	4	2.6
***Albuminuria***		
Negative	109	71.2
20mg/l	33	21.6
50mg/l	7	4.6
100mg/l	4	2.6
***Hematuria***		
Negative	142	92.8
+1	10	6.5
+2	1	0.7
Renal dysfunction*	48	31.4
***3 months follow up results (n = 48)***		
*Albuminuria (mg/l)*		
Negative	15	31.3
20mg/l	20	41.7
50mg/l	10	20.8
Unknown status	3	6.2
***eGFR < 60ml/min/1*.*73m2 at 3 months***	2	4.2
Kidney function at 3 months**		
Normalized kidney function	14	9.2
Renal dysfunction	31	20.3
Unknown status	3	2.0

*Albuminuria and/or eGFR<60ml/min/1.73m^2^

** Total number of children with HbSS (153) was used as a denominator

### Factors associated with the presence of renal dysfunction among children with SCD enrolled

Multivariate analysis was performed to relate demographic, clinical and laboratory characteristics associated with having renal dysfunction among study children. Blood in urine (OR 7.05 95% CI [1.36–37.70], p<0.020) and history of acute chest syndrome (OR 3.04, 95% CI [1.08–8.96], p = 0.044) were significantly associated with presence of renal dysfunction. Children with severe anemia were more likely to have renal dysfunction (OR 0.44, 95% CI [0.26–0.76] p = 0.003) while those with moderate to severe malnutrition defined as weight for height Z-scrore < -2 and -3SD respectively had a 5-fold higher chance of having Renal dysfunction (OR 4.92, 95% CI [1.29–18.84], p = 0.020). Additionally, those with positive urine CCA test were 7 times likely to have renal dysfunction compared to those with a negative test (OR 7.43, 95% CI [2.10–26.32] p<0.002). Five children reported to have a history of neurological deficits after presenting with hemiplegia but only 2 children had CT scan confirmed stroke, 2 children had a family history of unidentified kidney diseases and all of them had renal dysfunction. A detailed analysis of factors associated with renal dysfunction is as shown on “Table 4”.

**Table 4:** Factors associated with renal dysfunction among (153) children with HbSS.

Predictor	Unadjusted Odds ratio (95% confidence interval)	p-value	Adjusted Odds ratio (95% confidence interval)	p-value
***Positive Urine CCA test***	6.16 (2.31–16.41)	<0.001	7.43 (2.10–26.32)	0.002
***Hemoglobin level***	0.45 (0.28–0.73)	0.001	0.44 (0.26–0.76)	0.003
***Positive blood in urine***	8.60 (2.33–31.71)	0.002	7.05 (1.36–36.70)	0.020
***Nutritional status***				
***No malnutrition***	1		1	
***Mild malnutrition***	1.04 (0.40–2.70)	0.931	0.67 (0.19–2.39)	0.538
Moderate to severe malnutrition*	5.21 (1.76–15.46)	0.003	4.92 (1.29–18.84)	0.020
***History of acute chest syndrome***	2.43 (0.99–5.93)	0.051	3.04 (1.03–8.96))	0.044
***Female gender***	1.15 (0.52–2.54)	0.736	0.68 (0.25–1.86)	0.450
***Age in months***	1.01 (1.00–1.02)	0.043	1.00 (0.99–1.02)	0.582

*Moderate to severe malnutrition defined as WHO Weight/Height or BMI Z-score between negative 2 and negative 3SD to less than negative 3SD respectively.

## Discussion

In the region of Sub-Saharan Africa, which harbor more than 75% of the global SCD burden exist, other prevalent conditions with the potential to cause renal damage such as malaria, HIV infection, severe malnutrition and urinary tract schistosomiasis. Little data is available from this region on how the interaction between these common childhood conditions and SCD contribute to the occurrence of sickle cell nephropathy. Our study was conducted in northwestern Tanzania; a region with high prevalence of urinary schistosomiasis and sickle cell disease to estimate the prevalence of renal dysfunction in children with SCD and the effects of other renal insults prevalent in this region to the occurrence of renal dysfunction.

### Prevalence of renal dysfunction among children with sickle cell disease

At enrollment, 31.4% (48/153) of the children had renal dysfunction (defined as presence of albuminuria of >20mg/l, or eGFR <60ml/ml/min/1.73m^2^) and after three months follow up the prevalence of renal dysfunction dropped to 20.3%. No similar previous study had reported on the prevalence of renal dysfunction in children with SCD from this region but a study by Kayange et al in 2015 comparing the rate of renal dysfunction among HIV infected and noninfected children reported the prevalence of up to 41.8% among HIV negative patients raising concerns on the existence of other conditions leading to renal dysfunction such as SCD and urinary schistosomiasis[19]. The reported prevalence was higher than in our findings most likely because in their study, albuminuria and eGFR estimation was only performed once with no follow up whereas we observed a decreased prevalence after excluding transient proteinuria at 3 months follow up. Also, in their study the prevalence of schistosomiasis was 34.4% compared to13.7% (21/153) in our study which might have contributed to the high prevalence of renal dysfunction. The prevalence of 20.3% is actually likely to be an underestimate of renal dysfunction as children with SCD generally have hyperfiltration starting early in infancy which might lead into those with modest alteration in eGFR being classified as normal. A study by Saidia et al in 2012 conducted in Dar es Salaam Tanzania among 313 children attending the outpatient Sickle cell disease clinic reported that 14.7% of children most of them less than 10 years of age had renal dysfunction (defined as eGFR <60ml/ml/min/1.73m^2^). This prevalence was lower than our findings probably due to the fact that they only used eGFR and proteinuria to define renal dysfunction[20].The median age at diagnosis of SCD in our study was 30 months which is in line with other studies reported from sub Saharan Africa highlighting late diagnosis and delay to initiate comprehensive SCD care in areas where these services are available which could be the reason for the observed younger age at onset of Sickle cell nephropathy in Africa contrary to the relatively late onset of this complication in Europe and America[21,22]. Another study conducted in the Democratic Republic of Congo by Aloni et al, comparing renal functions among 67 non SCD and 65 SCD children reported 12.3% of children with SCD had renal insufficiency (defined as eGFR<80ml/min/1.73m^2^) which was lower compared to our findings and this can partly be explained by the difference in population prevalence of SCD and use of different urine strips for albumin estimation[23]. A study in the USA by Bodas et al in 2015, to investigate the prevalence of abnormal renal function among children with SCD reported 8.3% of enrolled children aged 3–18 years had eGFR<90ml/min/1.73m^2^, this study was a retrospective chart review and reported lower rates of abnormal renal functions compared to our study findings most likely as a result of the difference in the availability, accessibility and time at initiation of quality comprehensive Sickle cell disease care in developed countries such as newborn screening leading to early diagnosis, hydroxyurea therapy and chronic transfusion therapy which is rarely the case in most resource settings such as Tanzania[24].

At enrollment the prevalence of albuminuria in our study was 31.4% (48/153) declining to 20.3% (31/153) at 3 months follow up in line with other studies reporting the proteinuria prevalence range of 5% to 30% [25–30]. This decline in proteinuria should not be regarded as improvement in renal function for children with SCD as transient albuminuria has been reported in up to 7% of healthy children[31].

### Factors associated with renal dysfunction among children with SCD

Similar to other studies, our findings showed that renal dysfunction was more common in older children with a median age of 92 months (though this was not statistically significant (p = 0.582),) compared to younger age [26,29,32–34]. These findings were contrary to our initial hypothesis that coexistence of other renal insults such as repeated malaria infections, malnutrition and schistosomiasis would shift the age at onset of renal dysfunction in children with SCD to younger age though the cross sectional nature of our study does not bring us to this conclusion. To ascertain the impact of co-existing renal insults on the onset of renal dysfunction a longitudinal study with longer follow up period would be appropriate.

Low hemoglobin level was significantly associated with renal dysfunction in line with findings reported by McPherson et al 2011 in USA [29]. Similar to our findings, a study in Dar es Salaam Tanzania, reported anemia to be associated with low GFR and several other studies have shown significant association on the presence of albuminuria and low hemoglobin level[20,35].The association of renal dysfunction and severe anemia has mostly been linked to chronic hyperhaemolysis leading to vasculopathy as reported in a large multinational study of Sickle cell related renal dysfunction among West African children with sickle cell disease[36]. The association of anemia and albuminuria was also reported in a study from USA by Alvarez, et al in 2006 showing that early initiation of chronic transfusion was inversely related to the development of renal dysfunction[37]. Little is known on the contribution of anemia due to progressive renal dysfunction leading to declining erythropoietin, reduced oxygen carrying capacity and increased polymerization of HbSS in the renal medulla with accentuation of the renal dysfunction.

None of the clinical events like painful crisis and frequency of blood transfusion had association with renal dysfunction, similar to a study published by Saidia et, al in 2012[20]. History of acute chest syndrome and neurological manifestation (hemiplegia)were associated with renal dysfunction highlighting that similar pan vasculopathy mechanisms contribute to the occurrence of sickle cell nephropathy as reported from previous studies[24,29,32,38,39]. Five children with a neurologic deficit and those with acute chest syndrome also had severe anemia compared to the rest of the study population which might have added to the occurrence of renal dysfunction in the two groups.

Schistosomiasis infection was a strong predictor of renal dysfunction in our study and no studies have evaluated the contribution of schistosomiasis in relation to occurrence of renal dysfunction in children with SCD but studies in non-sickle cell subjects have reported schistosomiasis to cause kidney disease, albuminuria in particular[19,40,41].The use of urine circulating cathodic antigen does not allow the distinction of *Schistosoma haematobium* from *Schistosoma mansoni* but previous studies on the prevalence of the two parasites had reported a prevalence of 40–50% for S. haematobium and up to 2% for S.mansoni among school age children in this region[42]. Both S. haematobium and S.mansoni have been linked with development of variable histological patterns of glomerulonephritis through schistosome antigen immune complex deposition in the glomeruli[43]. Also, urinary schistosomiasis has been reported in some studies to cause urinary tract pathology from chronic bladder lesion caused by deposition of schistosome eggs marked by ultrasonographic lesions and high urine albumin to creatinine ratio [44,45]. The immune mediated glomerular lesions and urinary tract pathology caused by schistosome eggs on urinary bladder wall can be confirmed by renal biopsy and bladder wall ultrasonography respectively both of which were not performed in our study.

In our study, children with moderate to severe malnutrition were more likely to have renal dysfunction than their well-nourished counterparts, though it is unclear whether SCD predisposed them to both malnutrition and renal dysfunction or having renal dysfunction increased the risk for malnutrition. Similar to our study, low BMI was reported by King et al in 2011 to be associated with albuminuria, although the severity of malnutrition was not documented in their study[26].

Two of children enrolled in our study had a family history of kidney disease and both developed renal dysfunction, although it was unclear what types of renal diseases were in their families this might have increased the risk to renal dysfunction.

Several limitations affect the generalizability of our study findings such as the fact that our study was not a case control study to delineate factors leading to renal dysfunction among SCD as compared to non SCD children especially for transient albuminuria. Also not evaluating markers of hemolysis such as reticulocyte count, serum bilirubin level and lactate dehydrogenase (LDH) since chronic hemolysis has highly been associated with development of renal dysfunction by inducing vasculopathy[46]. We were also unable to assess the contribution of repeated malaria infection to renal injury in children with SCD after excluding all children with acute febrile illnesses to avert false positive albuminuria. The use of a semiquantitative micral test for urine albumin estimation compared to a laboratory based quantitative urine albumin to creatinine ratio (ACR) estimation probably affected the true prevalence of proteinuria especially in children above 10 years of age as previous studies had reported declining sensitivity in older children, this was minimized by a repeat test at three months follow up.

In conclusion; our study findings show that a significant proportion (20.3%) of children with SCD have renal dysfunction and other preexisting prevalent conditions like schistosomiasis and moderate to severe malnutrition potentially contribute to its development in this group of children calling for inclusion of measures to screen and prevent these co-morbidities during routine Sickle cell disease care.

We recommend further longitudinal case control studies to be conducted to evaluate the causal relationship between SCD and other prevalent renal insults on the onset and progression of renal dysfunction and the role of using noninvasive screening tools such as urine dipsticks for early detection of albuminuria and timely administration of treatments such as angiotensin converting enzyme inhibitors to prevent progression to renal dysfunction and ESRD.

## Supporting information

S1 Table This is the S1 table showing the estimated GFR by age range among children with SCD enrolled in our study. (PDF)

S2 Table This is the S2 table showing prevalence of renal dysfunction by age groups among children with SCD enrolled in our study. (PDF)

S1 File This is the data set on xcell spread sheet of clinical and laboratory details analyzed for children with SCD enrolled in our study. (XLSX)

## Abbreviations

CCA: Cathodic Antigen for Schistosomiasis
eGFR: estimated Glomerular Filtration Rate
ESRD: End Stage Renal Disease
HIV: Human Immunodeficiency Virus
SCD: Sickle cell Disease
