# Supplementary material for: Prevalence and factors associated with renal dysfunction among children with sickle cell disease attending the sickle cell disease clinic at a tertiary hospital in Northwestern Tanzania
Source: PLoS One. 2019 Jun 18;14(6):e0218024. doi: 10.1371/journal.pone.0218024 (PMC6581240; doi:10.1371/journal.pone.0218024)
Supplement: S1 Table — (PDF) [file pone.0218024.s001.pdf]

S1 Table. This is the S1 table showing the estimated GFR by age range among children with SCD enrolled in our study

eGFR range (mL/min/1.73m2)	Age range(years)			
	<3<br>N=40<br>Number(Percent)	3-6<br>N=34<br>Number (Percent)	6.1-9<br>N=34<br>Number(Percent)	9.1-12<br>N=45<br>Number(Percent)
30-59	3(7.5)	1(2.9)	0(0.0)	0(0.0)
60-89	16(40.0)	5(14.7)	1(2.9)	0(0.0)
90+	21(52.5)	28(82.4)	33(97.1)	45(100.0)
