# Supplementary material for: Prevalence and factors associated with renal dysfunction among children with sickle cell disease attending the sickle cell disease clinic at a tertiary hospital in Northwestern Tanzania
Source: PLoS One. 2019 Jun 18;14(6):e0218024. doi: 10.1371/journal.pone.0218024 (PMC6581240; doi:10.1371/journal.pone.0218024)
Supplement: S2 Table — (PDF) [file pone.0218024.s002.pdf]

S2 Table. This is the S2 table showing prevalence of renal dysfunction by age groups among children with SCD enrolled in our study

**Age group (years)**	<6<br>(n=73)	6-9<br>(n=35)	9-12<br>(n=45)
**Renal dysfunction**<br>n (%)	10(13.7)	10(28.6)	11(24.4)
**No Renal dysfunction**	63(86.3)	25(71.4)	34(75.6)

The prevalence of renal dysfunction in the age groups <6years, 6-9years and 9-12years were 13.7%, 28.6% and 24.4% respectively
